# Cracking the Code of Neuronal Cell Fate

**DOI:** 10.3390/cells12071057

**Published:** 2023-03-30

**Authors:** Giovanna Morello, Valentina La Cognata, Maria Guarnaccia, Velia D’Agata, Sebastiano Cavallaro

**Affiliations:** 1 Institute for Biomedical Research and Innovation, National Research Council (CNR-IRIB), 95126 Catania, Italy; 2 Section of Human Anatomy and Histology, Department of Biomedical and Biotechnological Sciences, University of Catania, 95124 Catania, Italy

**Keywords:** apoptosis, neurotrophic factors, survival, transcriptional analysis, drug targets, drug repurposing, functional enrichment, regulatory network, neurological disease

## Abstract

Transcriptional regulation is fundamental to most biological processes and reverse-engineering programs can be used to decipher the underlying programs. In this review, we describe how genomics is offering a systems biology-based perspective of the intricate and temporally coordinated transcriptional programs that control neuronal apoptosis and survival. In addition to providing a new standpoint in human pathology focused on the regulatory program, cracking the code of neuronal cell fate may offer innovative therapeutic approaches focused on downstream targets and regulatory networks. Similar to computers, where faults often arise from a software bug, neuronal fate may critically depend on its transcription program. Thus, cracking the code of neuronal life or death may help finding a patch for neurodegeneration and cancer.

## 1. Introduction

A central focus in neurobiology and neurology is the study of neuronal survival and apoptosis, two processes that guarantee the appropriate development of the central nervous system (CNS) and its homeostatic maintenance throughout adulthood. During development, in fact, the brain produces more neurons that will eventually survive, and the interplay of pro-apoptotic and pro-survival signal is essential for sculpting the nervous system. Following this developmental window, inappropriate activation of these mechanisms can contribute to the development of pathological conditions, such as brain tumors and neurodegenerative diseases [1,2,3].

Similar to computers, where faults often arise from malfunctioning software, neuronal fate critically depends on its programs. The ability of neuronal cells to promote or evade apoptotic cell death, in fact, does not depend by the expression or activity of a single gene/protein (hardware), but is regulated by a transcriptional program that is activated by different extracellular signals, including the absence or presence of neurotrophic factors (NFs) [4].

Although several studies have investigated the contribution of individual genes at the crossroad of apoptosis and survival signaling pathways, the complex and coordinated temporal transcriptional programs that orchestrate neuronal cell fate decisions remain mostly unknown. Thus “cracking” the code of neuronal life or death may not only provide new insights into the sophisticated cellular and molecular events underlying these processes but, more importantly, may help finding new treatment strategies for neuronal degeneration and cancer.

The advances in the field of omics technologies, together with the increase in knowledge and research of computational analysis and modeling of neuronal networks are now offering a systems biology-based approach to experimentally interrogate the complexity of transcriptional programs controlling neuronal apoptosis and survival and their malfunctions, offering a novel strategy to modulate these cell states. In this review, we will describe how portraits of whole-genome expression analysis coupled to reverse engineering of regulatory networks are beginning to decode the complex transcriptional programs underlying neuronal cell fate and the implication of their perturbation in human pathology, which are paving the way to innovative therapeutic approaches focused on downstream targets and regulatory networks.

## 2. Systems Biology Approaches to Explore the Transcriptional Programs Underlying Neuronal Apoptosis and Survival

Neuronal turnover is not a static process and both survival and apoptosis rely on transcription. The condition for *de novo* gene expression and the activation of a “suicide” transcriptional program during neuronal apoptosis was postulated about four decades ago [5,6,7,8,9]. During the years, multiple genes or genetic pathways have been implicated in apoptosis; however, our inability to experimentally resolve and interrogate the full spectrum of genes operating in distinct temporal domains and their collective behavior has hampered the progress in this field and we had to wait the development of high-throughput technologies to begin its exploration from a system’s biology perspective. The advent of omics technologies has, in fact, dramatically revolutionized the current understanding of the molecular mechanisms mediating neuronal survival and apoptosis and the transition between these two cellular states in a variety of experimental paradigms [10,11,12,13,14,15,16].

Despite *in vivo* studies better reproduce human pathology, neurons undergo apoptosis asynchronously and it is difficult to time the sequential transcriptional changes of different cell states. On the other hand, *in vitro* models, although somewhat artificial, allow the study of transcriptional changes in homogeneous neuronal populations undergoing synchronous cell states. Over the past decades, a large number of studies reported whole-genome expression analysis in multiple *in vitro* paradigms of neuronal apoptosis, including cerebellar granule neurons (CGNs), as well as cortical and hippocampal neurons, implying the existence of universal transcriptional mechanisms regulating neuronal cell death [9,10,11,12,13,14,15,16,17,18,19,20,21]. Among these experimental paradigms, CGNs represent, both *in vivo* and *in vitro*, the election model for examining the signal transduction mechanisms underlying neuronal apoptosis and survival [5,6,9,20,22,23,24,25,26,27,28,29]. CGNs are the most abundant type of neurons in the mammalian brain and can be cultured *in vitro* up to 98% homogeneity. *In vitro*, CGNs undergo rapid and synchronous apoptotic cell death within 24 h after removal of serum and lowering of extracellular potassium concentration from 25 to 5 mM [9,30]. This cell death paradigm presumably mimics the naturally occurring death of 20–30% of granule cells, which is essential for harmonizing their number with Purkinje cells between the third and fifth week postnatally, whereas its pathological counterpart causes an *in vivo* lesion model of deafferentation in adult rats cerebellar cortex [31,32]. Apoptosis of CGNs requires transcription and protein synthesis and the process becomes irreversible throughout the first 6 hours succeeding its induction. Before this “commitment point”, apoptosis of CGNs can be rescued following the activation of specific signal transduction pathways or by the administration of NFs [9,30]. Over the past 20 years, our research groups and others have begun to explore the transcriptional changes during the pre-commitment phase of apoptosis and its rescue by different NFs [6,9,10,22,23,33,34,35,36,37,38,39,40,41,42]. Taken together, these studies highlighted that, although distinct NFs exert their survival effects by binding specific receptors and activating a plethora of intracellular second messengers, their signaling pathways share striking similarities and are propagated by common transcriptional cascades, suggesting the existence of a conserved transcriptional program at the intersection of apoptosis and survival.

**Figure 1:**
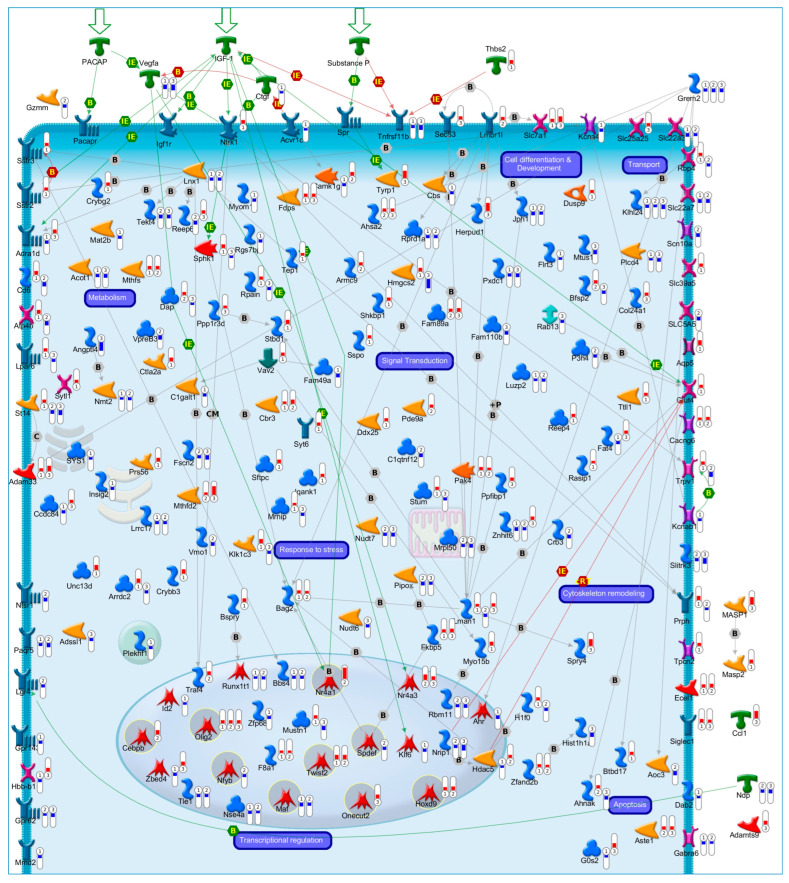
Transcriptional Profiles of Cell Fate Transitions Reveal Early Drivers of Neuronal Apoptosis and Survival. In our recent work [43], we identified a *core set* of 175 genes exhibiting a significant opposite expression trend during the early commitment phase (0.5 h, 1 h and 3 h) of CGNs apoptosis or its rescue by three NFs (Pacap, Igf1 and SP). The illustrative map shows the biological function and sub-cellular localization of the encoded proteins of *core set* genes. Significant gene expression changes are shown with “thermometer-like” figures. Numbers indicate time points: ① 0.5 h, ② 1 h, and ③ 3 h following induction of CGNs apoptosis and rescue by NFs. For each time-point, the upward thermometers (red) indicate gene transcripts up-regulated by NFs treatment, while downward thermometers (blue) indicate genes down-regulated. The pathway map was created using MetaCore Pathway Map Creator tool (GeneGo). Further explanations are provided at https://portal.genego.com/legends/MetaCoreQuickReferenceGuide.pdf. (accessed on 14 February 2023).

Beside the evidence of a conserved transcriptional program, the key drivers of neuronal fate transitions remained enigmatic until we shifted our attention to transcriptional regulatory networks. In a recent work, we investigated the dynamic transcriptomic changes occurring during the early commitment phase (0.5 h, 1 h and 3 h) following CGNs apoptosis or their rescue by three different NFs (Pacap, Igf1 and SP). Overlapping these temporal transcriptome profiles, we identified a *core set* of genes (175 genes) exhibiting opposite mRNA expression trends during neuronal apoptosis and NFs-mediated rescue (Figure 1) [43]. Of note, this *core set* included both rapid and delayed transcriptional changes in response to NFs rescue (Figure 1) [43]. In particular, *core set* genes encoding proteins involved in transcriptional activity, neuronal proliferation and differentiation, exhibited rapid and transient transcriptional activation, suggesting their primary role in the immediate response to NF-treatment (Figure 1) [43]. Among these were genes known for their contribution in the regulation of neuronal development and survival (i.e., *Pak4*, *Ntrk1*, *Twist2*, *Masp2*, *Hoxd9*, *Sstr2*, *Sstr3*, *Tep1*, *Tyrp1*) (Figure 1) [44,45,45,46,47,48]. The p21 activated kinase Pak4 plays a pivotal role in neuronal pathophysiology, promoting the activity of transcription factors involved in cell survival, such as Akt and Cepbp and, thus, contributing to a wide range of intracellular processes including cytoskeletal dynamics, neuronal development, axonal outgrowth and neuronal survival [49,50,51]. Down-regulated expression of *Pak4* has been associated with neurodegenerative disorders, including Amyotrophic Lateral Sclerosis and Parkinson’s disease, and it has been proposed as a therapeutic target against cancer and neurodegeneration [52,53,54,55]. Nerve growth factor receptor Ntrk1 promotes neuronal survival, proliferation and differentiation in neuronal populations, representing a clinically relevant target in neurology [56,57,58,59,60]. On the other hand, we also observed that delayed early response *core set* genes were, instead, enriched in different cellular processes, including cell adhesion, cytoskeleton organization, metabolic processes and oxidative damage, supporting their role as secondary effectors of the transcriptional program governing neuronal fate decision (Figure 1) [43].

Overall, these findings represent early portraits of the complex and coordinated temporal transcriptional programs underlying apoptosis and its rescue by NFs, further supporting the existence of a conserved transcriptional program governing neuronal life or death.

## 3. Apoptosis/Survival Switch and Human Diseases: At the Crossroads between Cancer and Neurodegenerative Diseases

As previously mentioned, while elimination of superfluous neuronal cells is essential for normal brain development, dysfunctions in the mechanisms leading to neuronal apoptosis or survival may play a role in different brain pathological conditions, including ischemia, cancer, neurodegenerative and neuropsychiatric disorders. In particular, defects in apoptotic cell death may promote development of brain cancers, while a significant increase in neuronal loss is associated with various psychiatric and neurodegenerative diseases, supporting these conditions may be considered the flip sides of the same coin and may derive from perturbations of the same regulatory mechanisms [4,61,62,63,64,65,66,67,68,69,70,71,72]. In light of these premises, it appeared evident that investigating molecular mechanisms regulating neuronal cell death or survival can be fundamental to explore the pathogenesis underlying pathological conditions and drive the development of targeted therapies.

**Figure 2:**
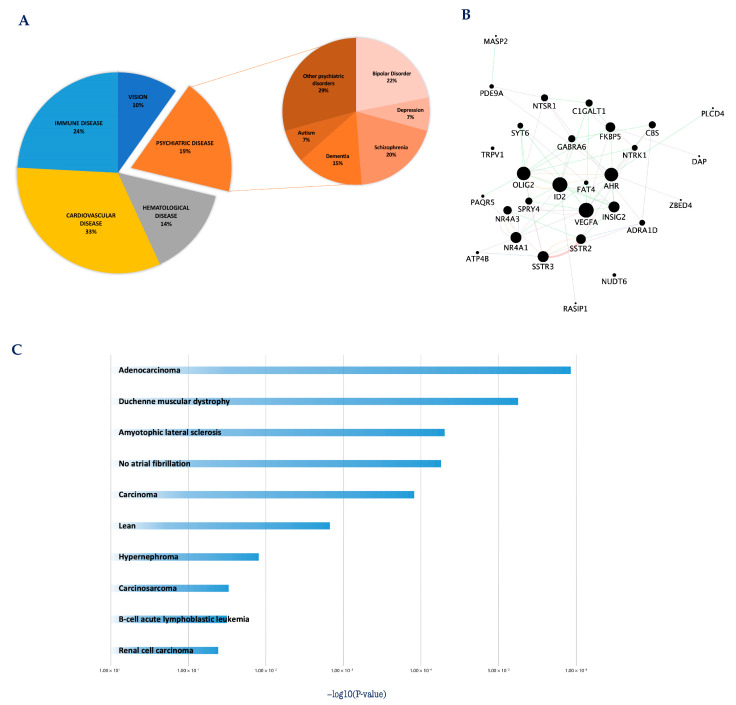
*Core set* genes are involved in the etio-pathogenesis of multiple cancer and neurological disorders. (**A**) Pie chart representation of *core set* genes implicated in human diseases. Disease enrichment analysis was performed with DAVID bioinformatics resources, including OMIM, KEGG DISEASE, and GAD catalogs. (**B**) The protein–protein interaction network of the 29 *core set* genes previously associated with cognitive/mental diseases. The network was built using the STRING website and visualized by Cytoscape (version: 3.8.2), by mapping the ‘degree parameter’ to node size. As the node size increased, the value of the connectivity degree of node genes increased. Differently colored ‘edges’ indicate the type of evidence supporting each interaction: dark purple: co-expression; light purple: physical interaction; light blue: co-localization; light green: shared protein domain; orange: predicted; grey: other. (**C**) Histogram of the most significantly enriched transcriptional signatures from iLINCS positively correlated with apoptotic CGN-related expression changes of *core set* genes. The significance of each disease related signature is represented by the enrichment scores value (−log10 (*p*-value)). For more details, please refer to the original work [43].

To better investigate this aspect, in our previous work, we evaluated the clinical relevance of *core set* genes involved with CGNs apoptosis and survival [43]. Disease enrichment analysis revealed the *core set* genes may be relevant in human pathology since genetic defects in most of them (121/175) have been associated with different human diseases (Figure 2A) [43]. Of particular interest is the significant association of a group of *core set* genes with cognitive/mental diseases (anxiety, attention deficit, schizophrenia, bipolar disorder, depressive syndrome and disruptive behavior disorders), supporting that a dysregulation of these apoptotic-related genes may contribute to the pathophysiology of these disorders (Figure 2A,B) [43,65,73,74,75]. Among these genes were key transcriptional regulators (e.g., *Ahr*, *Id2*, *Nr4a1*, *Nr4a3*, *Olig2*, *Zbed4*), sustaining previous evidence that several severe cognitive disorders are associated with alterations in transcriptional regulatory activity [76,77,78] (Figure 2B).

The potential contribution of *core set* genes in human pathology was further explored by directly matching the transcriptional changes of *core set* genes with disease-specific gene expression signatures included in the integrative Library of Integrated Network-Based Cellular Signatures (iLINCS, http://www.ilincs.org/ilincs/signatures/main) (accessed on 29 March 2023) [43]. This integrative web-based platform facilitates mining and re-analysis of user-submitted omics signatures in the context of a large collection of pre-computed disease signatures [79]. Our *in silico* analysis showed that *core set* expression patterns overlapped with transcriptional signatures associated with different human diseases, including various types of cancers and neurodegenerative disorders (Figure 2C), further supporting these two disease categories may derive from perturbations of the same regulatory mechanisms and can be considered the flip sides of the same coin [43]. Taken together, these data support the possibility that the early transcription changes associated with CGNs apoptosis and survival may be conserved in other different cells, tissues and species, thus sustaining the existence of a universal program governing cellular life-and-death processes.

Given the implication of *core set* genes in human pathology, our findings open the possibility to identify new or already existent therapeutics that are able to modulate their activity. To this regard, we performed a transcriptional signature connectivity analysis in iLINCS to explore repurposing drugs that could revert the expression of the *core set* genes during neuronal apoptosis, representing putatively therapeutically useful candidates [43]. iLINCS, in fact, also includes a comprehensive large-scale drug perturbation databases containing transcriptomic profiles of dozens of cultivated cell lines treated with thousands of chemical compounds serving as reference databases. By overlapping these drug perturbation signatures with the expression patterns of our apoptotic-related gene set, we identified candidate repurposable drugs that may reverse apoptosis (Table 1) [43]. Of note, almost all the perturbagens we have found are established neuroprotective entities [43]. Taken together, this evidence further supports the implication of the *core set* genes in human pathology and highlight the utility of their perturbation as a therapeutical strategy.

## 4. Cracking the Transcriptional Regulatory Programs of Neuronal Cell Fate May Orient New Therapeutic Strategies

Until today, different therapeutic strategies aimed at controlling neuronal apoptosis and survival have targeted the input (inducing signals) or output (executing machinery) underlying mechanisms, which can be considered the cellular “hardware”. Although many of these therapeutic strategies have been validated, most of them remain in the preclinical state because of lack of specificity and low efficacy [80]. An example is the use of NFs, whose therapy potential is hampered by the difficulty in delivering these proteins to the CNS and limiting their unwanted pleiotropic effects [81,82,83,84,85,86,87].

Similar to computers, where most of the problems commonly arise from buggy software, our neurons may also deal with malfunctions in the transcription regulatory program. Thus, cracking the code of neuronal fate may elicit novel pharmacological strategies, no longer oriented to the cellular hardware but, rather, the nuclear transcriptional regulatory mechanisms. Reconstruction of this cellular program by “reverse engineering” of gene regulatory networks (GRNs) poses great opportunities in systems biology [88,89,90,91,92,93,94,95,96,97] and allows to build accurate models of physiological and pathological processes, including those implicated in neuronal fate and development [98,99,100,101,102,103]. The impact of using these gene regulatory models to understand human diseases and find new treatments is profound, since they may allow to identify disease driver genes and promising biomarkers and therapeutic targets more efficiently and accurately [99,103,104,105,106].

**Figure 3:**
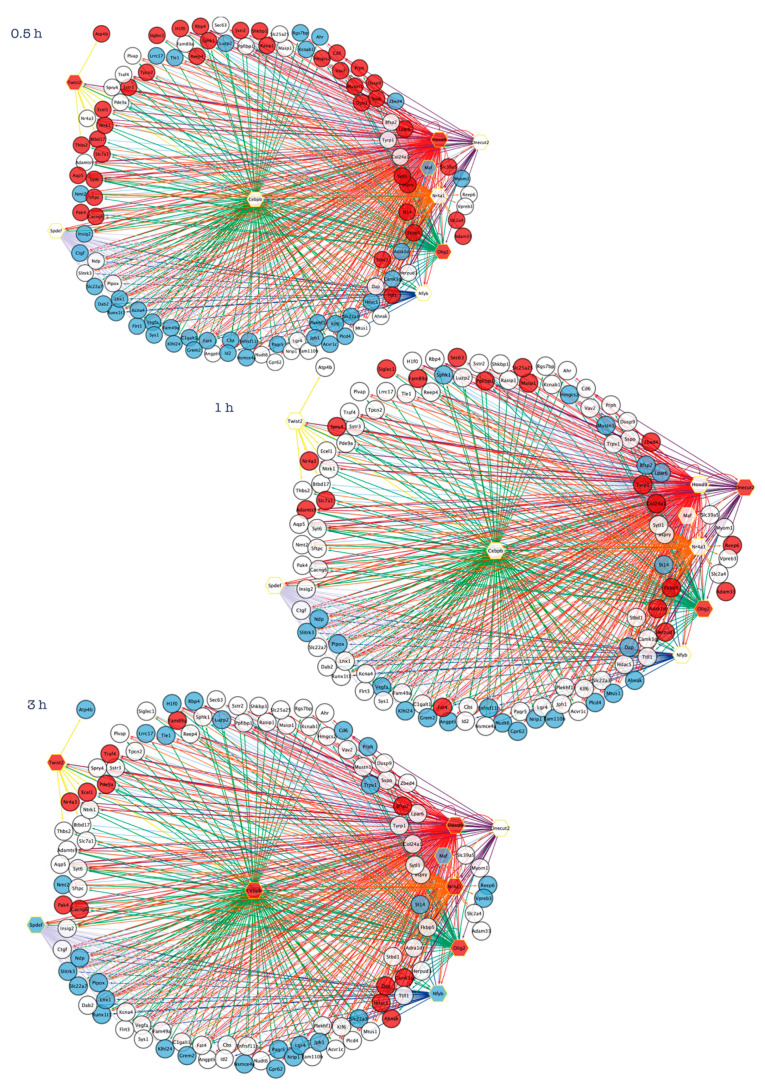
Reverse-engineering of transcriptional changes identifies key transcription factors at the intersection of neuronal apoptosis and survival. Inferring transcriptional gene regulatory networks of *core set* genes identify nine transcription factors (*Hoxd9*, *Maf*, *Nr4a1*, *Cebpb*, *Olig2*, *Onecut2*, *Spdef*, *Twist2*, *Nfyb*), which may act as upstream regulators of neuronal cell fate [43]. Transcription regulatory network analysis generated at each time point (0.5 h, 1 h, 3 h) following induction of apoptosis or rescue by NFs emphasizes how temporally distinct apoptosis and survival-inducing signals are orchestrated by the action of interconnected and temporally ordered TFs. Regulatory networks are visualized by Cytoscape and for each time-point the node color is consistent with the expression logFC of each gene: genes in blue are down-regulated by NFs treatment, while genes in red are up-regulated. Transcription factors are represented as hexagon nodes, while gene targets are represented as circle nodes. Regulons for each transcription factor are represented by different edge colors.

Recently, we applied a “reverse engineering” method to identify candidate upstream regulators of early transcriptional changes observed following induction of CGNs apoptosis and its rescue by NFs [43]. In particular, we performed an *in silico* analysis to predict transcription factors (TFs) whose binding motifs are enriched in the promoter regions of *core set* genes [43]. Our analysis revealed that temporally distinct modules of *core set* genes are regulated by the coordinated action of nine TFs (Hoxd9, Maf, Nr4a1, Cebpb, Olig2, Onecut2, Spdef, Twist2, Nfyb) that may act as upstream regulators of neuronal cell fate, converging apoptosis and survival-inducing signals in a highly interconnected and temporally ordered manner (Figure 3) [43]. In particular, these results showed a high degree of cross-regulation among the nine TFs as well as a common early (0.5 h and 1 h) and transient peak of transcription for the almost all TFs, with the exception of Onecut2 that was activated after 3 h following NF treatment (Figure 3). Of note, some of these transcription factors encode previously tested molecular/pharmacological targets and their exploitation may interfere with the early stages of the apoptotic/survival transcriptional program and represent novel therapeutic strategies [43]. In the following paragraphs, we will discuss these master regulators in light of their potential role as therapeutic targets for neurological disorders.

### 4.1. Homeobox D9 (Hoxd9)

Hoxd9 belongs to an evolutionarily conserved family of homeodomain-containing transcription factors that plays an important role during development of the central nervous system and continue to be expressed into adulthood [107,108,109,110]. Following their initial discovery, a substantial amount of information has been gained regarding the roles Hox genes play in various physiologic and pathologic processes, including brain cancer and neurological disorders, suggesting their molecular/pharmacological modulation as a potential strategy for therapies of complex human disorders [109,111,112]. The importance of *Hoxd9* as master regulator of neuronal cell fate is highlighted by its early transcriptional activation following NF-mediated rescue (Figure 1 and Figure 3) [43]. In accordance with these results, previous studies have demonstrated that Hoxd9 regulates the expression of several genes involved in neuronal apoptosis, displaying increased expression in unfavorable brain tumors, whereas its loss of function causes defects in axonal targeting and reduction in neural cell numbers, suggesting its utility as a potential therapeutic target at the crossroads between neurodegeneration and cancer [111,113,114,115]. To this regard, siRNA-induced silencing of *Hoxd9* gene has been already employed to induce apoptosis in different types of brain tumors, including neuroblastoma [116,117].

### 4.2. Nuclear Receptor 4A1 (Nr4a1)

Nr4a orphan nuclear receptor are a family of transcription factors that are rapidly and strongly up-regulated in response to a diverse range of signals, including growth factors, cytokines, membrane depolarization, oxidative stress and excitotoxic insults to the central nervous system, which up-regulate neuroprotective genes and improve neuronal survival [118,119,120,121]. Nr4a sub-family members are categorized as early-response genes, are robustly induced in the CNS by pathological stimuli such as ischemia, seizures and focal brain injury and have pleiotropic physiological roles, including maintenance of neuronal integrity, regulation of the density and distribution of spines and synapses, suppression of apoptosis and induction of pro-survival genes [119,120,122]. Among the key components of this TF family is Nr4a1, whose gene expression levels were reverted (up-regulated) following NFs-induced rescue effects and that we found involved in the transcriptional regulation of a large number of *core set* genes, including other TFs (*Maf, Nfyb and Spdef*), implicated in system development, regulation of apoptotic process, chemotaxis and metabolic process (Figure 1 and Figure 3) [43]. In particular, as an immediate-early gene, Nr4a1 modulates cell fate by controlling mitochondrial functions and synaptic activity in response to a variety of stressors and sensory stimuli [123,124]. Notably, a marked decrease of Nr4a1 was associated with a variety of neurological conditions, including Alzheimer’s and Parkinson’s diseases, and its pharmacological activation exerts neuroprotective, anti-inflammatory and pro-survival effects, proposing Nr4a1 as a potential therapeutic target for multiple neurological disorders [120,122,123,125,126,127,128,129,130,131,132,133,134]. Within this context, previous studies have provided evidence for the implication of nuclear receptors (i.e., Nr4a1 and Nr4a3) in schizophrenia and bipolar disorders, demonstrating that down-regulated expression levels or sequence variations correlate with increased susceptibility to these cognitive disorders (Figure 2) [126,127,135,136].

### 4.3. Musculoaponeurotic Fibrosarcoma (Maf)

Maf (also known as c-Maf or v-Maf) is a member of a large group of b-Zip proteins that form a complex regulatory network, acting either as transcriptional activators or repressors of multiple cellular genes involved in immune response, apoptosis as well as neuronal outgrowth, maintenance and differentiation [19,109,122]. Despite little is known about the specific role of Maf in neuronal apoptosis and degeneration, recent reports suggest that over-expression of this gene can cause cell death probably through a p53-mediated signaling [122,137]. In agreement with these results, we found an early and transient peak of transcription for *Maf* following induction of CGNs apoptosis, while its expression decreased following treatment with NFs (Figure 1 and Figure 3) [43]. As other oxidative stress reactive proteins, Maf has been implicated in various neurological disorders, including Alzheimer’s and Parkinson’s diseases, and its role is emerging as a novel target in the treatment of these disorders [138,139,140].

### 4.4. CCAAT Enhancer Binding Protein Beta (Cebpb)

*Cebpb* encodes a basic-leucine zipper transcription factor that plays pivotal roles in development and synaptic plasticity of the nervous system, regulating the expression of genes involved in cell differentiation, neuronal development, immune response, neuronal apoptosis and metabolism [141,142,143,144,145,146]. In accordance with our study showing a transient activation of *Cebpb* during NFs-mediated rescue from apoptosis [43], previous studies demonstrated that up-regulation of this TF in rat primary cortical and cerebellar neuronal cultures plays neuroprotective and antiapoptotic effects, while its reduced neuronal levels may represent a pathogenic factor in neurodegenerative disorders, including Alzheimer’s and Parkinson’s diseases, supporting the potential of Cebpb as a pharmacological target in brain injury and neurodegenerative disorders (Figure 1 and Figure 3) [141,147,148,149,150,151,152].

### 4.5. Oligodendrocyte Transcription Factor2 (Olig2)

Among the enriched TFs in up-regulated core set genes, we identified the basic-helix-loop-helix (bHLH) transcription factor Olig2 that plays a key role in directing cell fate choices, promoting cell proliferation and controlling CNS development [43,153,154]. Several studies, in fact, have demonstrated that activation of Olig2 in response to different NFs (e.g., FGF, GDNF and PDGF) exerts protective and pro-survival effects in multiple neuronal types [155,156,157,158]. According with these findings, we observed an increased expression of Olig2 during CGNs rescue by Pacap, Igf1 and SP (Figure 1 and Figure 3) [43]. Despite the role elicited by Olig2 in the adult cerebral cortex under pathological conditions is not yet known, its reduced expression seems to switch cell fate from differentiation to death, contributing to the development of psychiatric disorders and acute/chronic neurodegenerative diseases, including Alzheimer’s disease and Amyotrophic Lateral Sclerosis, while its increased expression has been associated with different brain tumors, supporting its potential utility as therapeutic target for the treatment of both cancer and neurodegeneration [159,160,161,162,163,164,165,166,167,168,169,170,171]. Of note, several evidence showed that Olig2 deficiency as well as the presence of rare genetic polymorphisms in this gene (i.e., rs1059004) may represent risk factors for cognitive disorders and schizophrenia, through an effect on neuroanatomical connectivity (Figure 2) [159,164,172,173,174,175,176].

### 4.6. One Cut Homeobox 2 (Onecut2)

Onecut2 encodes a member of a family of transcription factors that function as transcriptional activators controlling cell differentiation and survival, as well as oxidative defense signaling, and that have only been recently proposed as regulators of neuronal differentiation [177,178,179,180,181,182,183,184]. Showing a consistent increased expression of this TF in CGNs during NF-mediated rescue, our results support the importance of Onecut2 for neuronal survival (Figure 1 and Figure 3) [43]. Although the expression of this factor is dysregulated in different types of tumors or following hypoxic insult to neurons, future studies are needed to further investigate the utility of Onecut2 as a target for brain tumors or neurodevelopmental disorders.

### 4.7. SAM Pointed Domain Containing ETS Transcription Factor (Spdef)

Spdef is an ETS (E26 transformation-specific) transcription factor, highly expressed in the prostate but also expressed in the brain and liver, where it regulates cellular differentiation, proliferation, cell-cycle control and apoptosis [185]. We observed an increased expression of *Spdef* throughout the time-course of CGNs apoptosis and its down-regulation following NFs treatment (Figure 1 and Figure 3) [43]. This evidence supports previous studies proposing its role as a tumor suppressor in various types of cancers [186,187]. In addition, altered expression of SPDEF has been found in Alzheimer’s disease patients and animal models, while a recent association was found between blood-based Spdef methylation and stress response, altered dopaminergic neurotransmission and increased vulnerability to substance abuse, suggesting its role as a biomarker for these pathological conditions [188,189,190,191].

### 4.8. Nuclear Transcription Factor Y Subunit Beta (Nfyb)

According to our recent findings showing increased expression of Nfyb during CGNs apoptosis [43], previous works demonstrated that its induction promotes neuronal apoptosis via the proapoptotic protein Bim and the activation of the p53 signaling pathway (Figure 1 and Figure 3*)* [192,193,194]. Nfyb regulates transcription of several genes that are related to cell cycle and its alterations contribute to neurodegeneration and apoptosis [195,196].

### 4.9. Twist Family BHLH Transcription Factor 2 (Twist2)

Twist2 is a highly conserved member of the Twist subfamily of basic Helix-Loop-Helix (bHLH) transcription factors that have been implicated in the transcriptional regulation of developmental programs in multiple cell lineages, and that are known to play important roles in cell migration, inflammation, protection of cells from apoptosis, and cellular response to oxidative stress [197,198,199]. According with the anti-apoptotic, anti-oxidative and anti-inflammatory effects of this TF, we observed its increased expression following NF-induced neuronal apoptotic rescue (Figure 1 and Figure 3) [43]. From a clinical point of view, Twist2 is upregulated in a variety of cancers, including glioma and neuroblastoma [181]. In addition, Twist2 dysfunctions are associated with human pathological conditions characterized by oxidative stress-induced neuronal death, supporting its potential role as therapeutical target for cancer and neurological diseases [199,200,200,201,202,203].

## 5. Conclusions

In this review, we highlighted how whole-genome gene expression analysis coupled to reverse engineering of gene regulatory networks are beginning to decode the complex transcriptional programs underlying neuronal apoptosis and survival [43]. This new experimental approach may foster an innovative pharmacology no longer oriented to influence the cellular hardware but focused on its regulatory transcriptional program.

## Figures and Tables

**Table 1:** List of the most enriched “repurposable” drug candidates with a potential to reverse apoptotic CGNs transcriptomic signature.

	Perturbation	*p*-Value	Perturbation	*p*-Value
Inflammation & Immunologic disorders	Indomethacin	3.10 × 10^−5^	Nystatin	3.24 × 10^−5^
	Dipyrone	1.07 × 10^−4^	Tranilast	3.41 × 10^−5^
	Sulfanilamide	1.12 × 10^−4^	Cyproheptadine	1.62 × 10^−2^
	Rifabutin	2.34 × 10^−4^	Rapamycin	1.66 × 10^−6^
	Allopurinol	8.01 × 10^−6^	Tacrolimus	5.22 × 10^−5^
	Necrostatin	5.29 × 10^−5^	Theophylline	3.80 × 10^−5^
Cancer	Tozasertib	2.08 × 10^−5^	Tyrphostin	1.54 × 10^−15^
	L-Sulforaphane	1.00 × 10^−4^	Tanespimycin	7.69 × 10^−5^
Psychiatric disorders	Tianeptine	8.71 × 10^−5^	Moclobemide	2.22 × 10^−4^
	Amitriptyline	7.97 × 10^−6^	Rolipram	2.15 × 10^−4^
	Nortriptyline	8.62 × 10^−6^	Azacyclonol	1.55 × 10^−2^
	Bupropion	1.57 × 10^−5^	Piracetam	4.11 × 10^−5^
	Roflumilast	1.84 × 10^−5^	Promazine hydrochloride	5.32 × 10^−4^
	Citalopram	3.85 × 10^−5^	Phenotiazine	1.09 × 10^−4^
	Iproniazid	7.33 × 10^−5^	Clozapine	1.35 × 10^−4^
	Doxepin	1.94 × 10^−4^	Diazepam	8.10 × 10^−5^
Epilepsy	Lamotrigine	1.68 × 10^−4^	Ethosuximide	1.07 × 10^−4^
Cardiovascular diseases	Enaplapril	2.75 × 10^−4^	Atorvastatin	1.03 × 10^−6^
	Nifedipine	2.79 × 10^−4^	Nicergoline	1.91 × 10^−4^
Other	Monorden/Radicicol	6.94 × 10^−3^	5-Nonyloxytryptamine	2.72 × 10^−4^
	Purmorphamine	1.80 × 10^−4^	Parthenolide	4.11 × 10^−5^
	Pifithrin	2.95 × 10^−30^		

## Data Availability

Not applicable.
